# Porous and Tough Polyacrylamide/Carboxymethyl Cellulose Gels Chemically Crosslinked via Cryo-UV Polymerization for Sustained Drug Release

**DOI:** 10.3390/gels11060453

**Published:** 2025-06-13

**Authors:** Duangkamon Viboonratanasri, Daniel Rudolf King, Tsuyoshi Okumura, Mohamad Alaa Terkawi, Yoshinori Katsuyama, Milena Lama, Tomoki Yasui, Takayuki Kurokawa

**Affiliations:** 1 Graduate School of Life Science, Hokkaido University, Sapporo 001-0021, Japan; viboonratanasri.duangkamon.j9@elms.hokudai.ac.jp; 2 Faculty of Advanced Life Science, Hokkaido University, Sapporo 001-0021, Japant.okumura@sci.hokudai.ac.jp (T.O.); y.katsuyama@sci.hokudai.ac.jp (Y.K.); tyasui@sci.hokudai.ac.jp (T.Y.); 3 Faculty of Medicine and Graduate School of Medicine, Hokkaido University, Sapporo 060-8638, Japan; materkawi@med.hokudai.ac.jp; 4 Institute for Chemical Reaction Design and Discovery (WPI-ICReDD), Hokkaido University, Sapporo 001-0021, Japan; lama@sci.hokudai.ac.jp

**Keywords:** carboxymethyl cellulose, polyacrylamide, porous, toughness, drug delivery, cryogelation

## Abstract

While carboxymethyl cellulose (CMC)—a biocompatible and water-soluble cellulose derivative—holds promise for biomedical applications, challenges remain in synthesizing CMC-based hydrogels with covalent crosslinking through free radical polymerization without requiring complex, multi-step processes. In this study, we introduce a facile one-pot strategy that combines CMC with acrylamide (AAm) under cryogelation and low-intensity UV irradiation to achieve covalent bonding and a high polymerization yield. The resulting polyacrylamide/carboxymethyl cellulose (PAAm/CMC) porous gels were systematically evaluated for their chemical, physical, thermal, and drug-release properties, with a focus on the effects of AAm concentration and polymerization temperature (frozen vs. room temperature). Notably, the cryogel synthesized with 2.5 M AAm (PC2.5) exhibited significantly enhanced mechanical properties—that is, an 8.4-fold increase in tensile modulus and a 26-fold increase in toughness—compared with the non-cryo gel. Moreover, PC2.5 demonstrated excellent cyclic compression stability in water and phosphate-buffered saline (PBS), with less than 10% reduction in modulus after 100 cycles. These increases in the mechanical properties of PC2.5 are attributed to the formation of macropores with high polymer density and high crosslinking density at the pore walls. PC2.5 also showed slower drug release in PBS and good cytocompatibility. This study presents a simplified and efficient route for fabricating mechanically robust, covalently crosslinked PAAm/CMC cryogels, highlighting their strong potential for biomedical applications in drug delivery systems.

## 1. Introduction

Carboxymethyl cellulose (CMC)—a water-soluble cellulose derivative with carboxy groups—has emerged as a promising material due to its anionic linear polymer structure, high water adsorption capacity, and excellent biocompatibility. These attributes have propelled CMC’s utilization in various applications in the biomedical, agricultural, pharmaceutical, and food industries [1,2]. Typically, CMC-based hydrogels are prepared via physical or chemical crosslinking. Physical crosslinking—which relies on electrostatic, ionic interactions, or hydrogen bonding—offers simplicity but often results in hydrogels with poor mechanical properties [3]. Conversely, chemical crosslinking—employing metal ions, radiation, or chemical crosslinkers—enables the formation of robust hydrogels but may introduce toxicity concerns or require complex procedures [4,5,6]. Notably, CMC hydrogels often exhibit poor mechanical properties, prompting researchers to explore composite hydrogels incorporating other polymers (both synthetic and natural), such as gelatin, chitosan, polyvinyl alcohol, poly(N-isopropylacrylamide), and polyacrylamide (PAAm) [7,8,9]. PAAm, a hydrophilic polymer derived from acrylamide, is an attractive candidate for enhancing the mechanical integrity of CMC-based hydrogels. Its excellent swelling properties, thermal stability, biocompatibility, and facile crosslinking with N, N′-methylenebis(acrylamide) (MBAA) facilitate the formation of robust hydrogels with controlled crosslinking densities [10]. Furthermore, the hydrogen bonding between -NH_2_ in PAAm’s amide groups and CMC’s carboxy groups contributes to enhanced mechanical properties, particularly in terms of rapid and complete stress recovery [11]. However, the conventional synthesis of PAAm/CMC hydrogels often involves multi-step processes and requires complex equipment to generate the CMC network, hindering their practical application. For instance, Cheng and coworkers fabricated a PAAm/CMC hydrogel using two types of crosslinking agents—MBAA (PAAm crosslinker) and ferric chloride (CMC crosslinker)—and a two-step polymerization process [12]. In another study, PAAm/CMC hydrogels were fabricated by gamma ray irradiation with and without a crosslinker [13]. However, this approach requires specialized facilities. Godiya’s, Mohy Eldin’s, and Omer’s groups attempted to fabricate grafted CMC with acrylamide-based superabsorbent hydrogels using MBAA and ammonium persulfate (APS), with potassium persulfate acting as an initiator via free radical polymerization [14,15,16]. They also prepared CMC/PAAm hydrogels via free radical polymerization of AAm into the CMC network; however, they did not provide evidence of CMC hydrogel formation using their methods. The formation of a covalent bond between CMC and AAm is impossible; thus, the synthesis of PAAm/CMC hydrogels with high yield, a high number of covalent bonds, and facile fabrication with high mechanical performance is an area of interest for further study.

Cryogelation has been widely employed to prepare porous hydrogels with interconnected macropores that facilitate nutrient diffusion, enhance mechanical toughness, and improve drug delivery performance [17,18,19,20]. During freezing, the cryoconcentration of solutes in the unfrozen liquid microphase can accelerate free radical reactions by increasing the local concentrations of monomers, polymers, crosslinkers, and initiators [20,21]. While the use of UV irradiation during cryogelation has been reported for other cellulose derivatives [19,22,23], its application in unmodified CMC to achieve covalent bonding with AAm monomers remains underexplored.

In this study, we build upon these concepts and propose a one-pot synthesis approach for fabricating PAAm/CMC porous gels via cryogelation combined with low-intensity UV-initiated free radical polymerization. By avoiding chemical modification of CMC or the use of organic solvents, and by operating under mild UV conditions, this method aims to provide a simplified and efficient route for fabricating biocompatible, covalently crosslinked porous gels. The influences of AAm concentration and polymerization temperature (cryogelation vs. room temperature) on the structural, mechanical, and drug release properties of gels are systematically investigated. The potential for use of the resulting gels in sustained drug delivery is also assessed. This study aims to provide a practical and approachable method for preparing multifunctional PAAm/CMC cryogels with potential for biomedical applications in drug delivery systems.

## 2. Results and Discussion

In this study, we achieved facile one-pot preparation of porous gels using AAm, CMC, MBAA (crosslinker), and 2-oxoglutaric acid (α-keto; initiator) as starting materials, under cryogelation with the UV irradiation polymerization method. Conventional gelation (polymerization at room temperature) occurs through the formation of intermolecular bonds in a homogeneous solution. For cryogelation, the moderate freezing process occurs uniformly, allowing for the formation of an interconnected network of ice crystals that serve as a template for pores, together with a highly concentrated monomer solution. Subsequently, polymerization and crosslinking reactions in nonfrozen liquid microphase domains of the cryogelation system result in the formation of a dense polymer hydrogel acting as the pore walls [19,20,21]. Furthermore, this study investigated the key parameters influencing the free radical polymerization reaction in the synthesis of CMC-based hydrogels, as summarized in Table S1. From these results, we investigated five parameters: polymerization temperature, UV irradiation, CMC concentration, and presence of a crosslinker and an initiator. Notably, a high concentration of substances enabled high radical generation under low-intensity UV light—likely due to the reduced intermolecular distances between radicals without water interference—thus facilitating polymerization [24]. This observation aligns with the gel formation of CMC-based hydrogels prepared at room temperature (Figure S1). Many researchers have attempted to create CMC-based hydrogels via free radical polymerization using various external energy sources, as detailed in Table 1. High-energy approaches, such as gamma ray radiation or ionizing radiation, typically require oxygen removal, a vacuum system, or operation under low pH conditions to achieve high polymerization yields. Although Fekete’s team formed a CMC network using gamma ray radiation with MBAA under air conditioning, the yield remained low. Alternatively, UV irradiation was applied as an energy source; however, this necessitated the modification or functionalization of CMC in organic solvents. MBAA effectively acted as a crosslinker of both CMC and AAm molecules. Furthermore, cellulose derivatives can generate macroradicals using UV light under cryogelation with the aid of a photoinitiator and subsequent recombination of the macroradicals to form gel networks [22]. Consequently, cryoconcentration significantly enhances radical formation and accelerates the polymerization rate, even when the precursor solution is prepared with relatively low initial chemical content [19,25]. Conversely, preparing CMC-based hydrogels at room temperature using high initial concentrations is challenging due to the resulting high viscosity of the mixture.

We used the following experimental conditions: UV irradiation power and irradiation time, as well as MBAA and α-keto concentrations. This study included five types of samples: non-cryogel (NP), CMC porous gel (CMC gel), PAAm porous gel (PAAm gel), dual-step synthesis porous gel (DSPC), and polyacrylamide/carboxymethyl cellulose porous gel (PC). The results suggested that the NP prepared using a mixture of AAm, low CMC content, MBAA, and α-keto was not an interpenetrating polymer network (IPN), as CMC could have formed an individual network under room temperature conditions. Additionally, CMC could have formed physical interactions, including H-bonding and entanglement [14]. For PC gels, the system exhibits a high potential for covalent interactions, including linkages between CMC and MBAA, PAAm and MBAA, and CMC and PAAm due to highly generated radicals under cryogelation. The porous gels prepared using one-pot synthesis could be copolymers. Finally, DSPC was the porous gel prepared via two-step fabrication and was an interpenetrating polymer network (IPN). Furthermore, the two-step synthesis of DSPC generated more chemical waste than the one-step method. This was primarily due to the disposal of the large volume of unreacted AAm monomer solution used in the soaking step. Conversely, the one-pot synthesis produced significantly less waste, largely owing to its high yield (approaching 100%), which minimized the amount of residual monomer requiring disposal.

### 2.1. Chemical Characterizations

**Figure 1:**
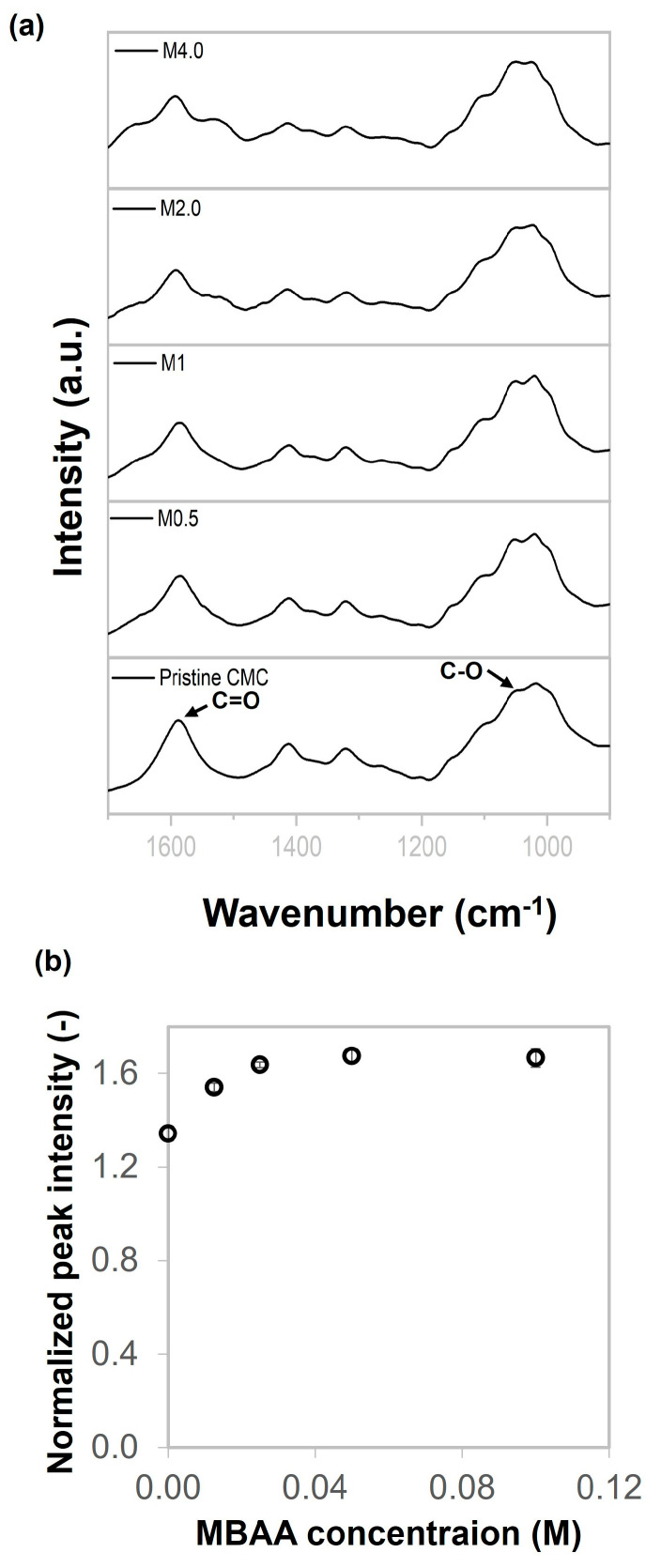
(**a**) FTIR spectrum of pristine CMC and CMC gels prepared with various MBAA contents, and (**b**) peak intensity at 1052 cm^−1^ normalized to the peak intensity at 1588 cm^−1^ as a function of MBAA concentrations.

The formation of chemical linkages between CMC and MBAA was confirmed using attenuated total reflectance Fourier-transform infrared (ATR-FTIR) spectroscopy. Figure 1 shows the FTIR spectra of pure CMC and CMC gels prepared with varying concentrations of MBAA, ranging from 0.5 to 4.0 mol%, named M0.5, M1.0, M2.0, and M4.0. The peak observed at 1588 cm^−1^ is assigned to the C=O stretching vibration. C-O-H in the anhydroglucose ring can generate radicals, and this carbon may potentially bond with other molecules [15,32,33,34]. Additionally, there was an important peak at 1052 cm^−1^, which was assigned to the CH-O-CH_2_ group resulting from the chemical reaction between the hydroxyl group located in anhydroglucose and the π-bond of MBAA. Therefore, the 1052 cm^−1^ peak was monitored to determine the extent of crosslinking between CMC and MBAA. The normalized peak intensity was associated with MBAA content, as shown in Figure 1b. The results showed that the normalized peak intensity value of M0.5 increased by about 1.14 times compared with pristine CMC. Then, the normalized peak intensity value rose continuously as MBAA was added until 2 mol%. The normalized peak intensity value remained relatively stable from 2.0 to 4.0 mol%. This behavior suggests that most reactive sites in the CMC had already reacted by 2.0 mol% MBAA. These results indicate that MBAA effectively crosslinked with CMC during the freezing and UV irradiation process.

### 2.2. Morphologies of CMC Gel, NP, PAAm Gel, DSPC, and PC Gels

**Figure 2:**
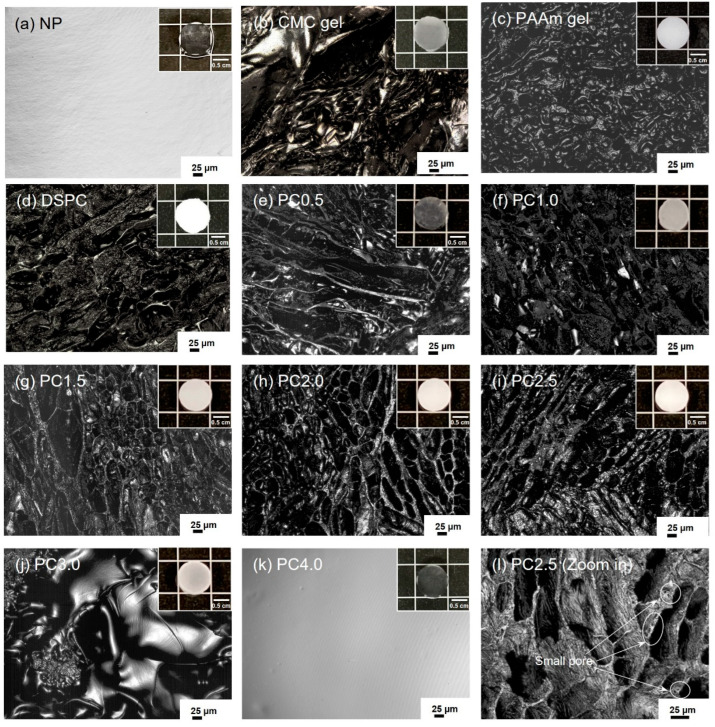
Morphologies of (**a**) NP, (**b**) CMC gel, (**c**) PAAm gel, (**d**) DSPC, (**e**–**k**) PC gels with various concentrations of AAm (0.5–4.0 M), and (**l**) zoomed-in view of PC2.5. Scale bars are 25 μm.

The morphologies of swollen hydrogels were observed using 3D laser scanning microscopy, as shown in Figure 2. The results showed that their pores were irregularly shaped, non-unidirectional, porous, and interconnected, resulting from the growth of ice crystals from frozen water [19,35,36]. Furthermore, all the porous gels were tested by squeezing, which resulted in the water immediately leaking out of the porous gels. The porous gels could reabsorb the water, as shown in Video S1. PC gels were prepared with various AAm concentrations ranging from 0.5 to 4.0 M, denoted as PC0.5, PC1.0, PC1.5, PC2.0, PC2.5, PC3.0, and PC4.0.

The average pore length of all porous gels (CMC gel, PAAm gel, DSPC, and PC gels) was 54–122 µm. In addition, PC2.5 included both small (as shown in Figure 2l) and large pores, with pore lengths ranging from 2.6 to 205 µm. The porous gel prepared with AAm at 3.0 M (PC3.0) had a pore length of approximately 13 μm. These results confirm that the porous gels were supermacropore hydrogels because the pores were larger than 1 μm, which is suitable for human cell culture [37]. Notably, the macropores of NP and PC4.0 could not be observed.

### 2.3. Mechanical Properties of CMC Gel, PAAm Gel, NP, DSPC, and PC Gels

**Figure 3:**
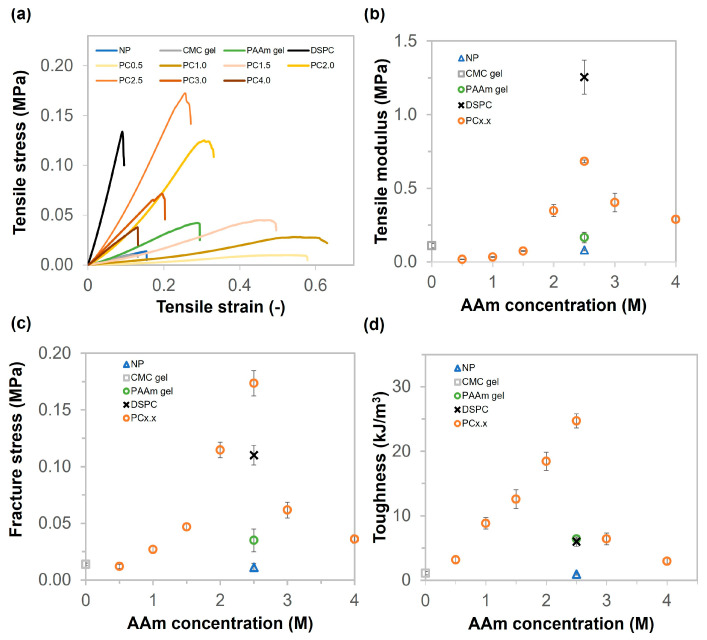
Tensile properties of NP, CMC gel, PAAm gel, DSPC, and PC gels (various AAm concentrations from 0.5 to 4 M). (**a**) Stress–strain curves of NP, CMC gel, PAAm gel, DSPC, and PC gels (various AAm concentrations from 0.5 to 4 M). (**b**–**d**) Tensile modulus, fracture stress, and toughness of NP, CMC gel, PAAm gel, DSPC, and PC gels (various AAm concentrations from 0.5 to 4.0 M).

The mechanical properties of the gels were measured using tensile and compression tests after swelling. The tensile results of swollen gels are shown in Figure 3. The tensile behavior of the hydrogel is described by tensile stress vs. tensile strain in Figure 3a. The tensile modulus values of NP, CMC gel, and PAAm gels were 0.08, 0.11, and 0.17 MPa, respectively (Figure 3b). The mechanical properties of the gels increased when the gels had pores and were prepared using a combination of synthetic monomers and natural polymers. The highest tensile modulus was that of DSPC (approximately 1.25 MPa, which was twice that of PC2.5). The tensile modulus values of PC gels continuously increased from 0.5 to 2.5 M of AAm concentration, ranging from 0.02 to 0.68 MPa. The tensile modulus of PC2.5 was about 8.4 times higher than that of NP. It is possible that the porous structure, high-density polymer matrix, and high crosslinking density in the pore walls increased the mechanical properties of the porous gels compared with non-cryo ones; as a result, the porous gels were stiffer than the non-cryo gel. When the AAm concentrations were 3.0 M and 4.0 M, the tensile modulus values decreased significantly to 0.40 MPa and 0.29 MPa, respectively. It is possible that PC3.0 has fewer pores, whereas PC4.0 does not have macropores. The tensile modulus of all samples ranged from 0.1 to 1.25 MPa. These values fall within the range of Young’s modulus of natural soft tissues and organs, which spans from 0.1 kPa to 1.0 MPa [38]. Therefore, all hydrogel samples can be considered as candidates for biomedical engineering materials. The fracture stress and toughness values of NP, CMC gel, PAAm gel, DSPC, and PC gels are shown in Figure 3c,d. The fracture stress and toughness values of NP and CMC gels were around 0.01 MPa and 0.96 kJ/m^3^ and 0.01 MPa and 1.1 kJ/m^3^, respectively. The PAAm gel showed higher fracture stress and toughness than NP and CMC gels, which were 0.03 MPa and 6.4 kJ/m^3^, respectively. The fracture stress values of PC gels increased continuously from 0.01 to 0.17 MPa as the monomer concentration increased from 0.5 to 2.5 M, demonstrating that PC2.5 was very ductile. PC2.5 exhibited greater toughness than DSPC due to the difference in MBAA content. The cryogels formed at low initial concentrations were mechanically weak; meanwhile, increasing the initial concentration enhanced their mechanical strength [39]. However, the fracture stress and toughness decreased dramatically for the PC gels with monomer concentrations above 3.0 M. Furthermore, as-prepared PC2.5 was approximately four times stiffer than as-prepared NP, as shown in Figure S2. It is possible that the highly dense polymer matrix and CMC bonding could affect the mechanical properties, especially in terms of toughness.

**Figure 4:**
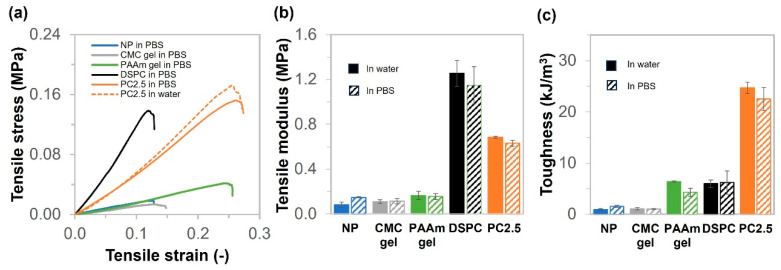
Tensile properties of NP, CMC gel, PAAm gel, DSPC, and PC2.5 in PBS. (**a**) Stress–strain curves of NP, CMC gel, PAAm gel, DSPC, and PC2.5. (**b**) Tensile moduli of NP, CMC gel, PAAm gel, DSPC, and PC2.5 under water and PBS. (**c**) Toughness of NP, CMC gel, PAAm gel, DSPC, and PC2.5 under water and PBS.

In the case of biomedical engineering applications, the materials were used in a saline solution; thus, we conducted studies on the mechanical properties under PBS conditions. After changing the condition from pure water to PBS for immersion of the gels, the mechanical properties of the five types of samples (NP, CMC gel, PAAm gel, DSPC, and PC2.5) were characterized by tensile test under humid conditions to prevent drying. The stress–strain curves, tensile moduli, and toughness are shown in Figure 4. The lowest and highest moduli were for CMC gel (0.11 MPa) and DSPC gel (1.15 MPa). However, the highest toughness was obtained with PC2.5, at about 26.6 kJ/m^3^. The mechanical properties of NP and CMC gels immersed in PBS increased slightly, when compared with those in water. Conversely, the mechanical properties of PAAm gel, DSPC, and PC2.5 were not significantly different after changing the solution from water to PBS. Additionally, both the tensile modulus and toughness of NP in PBS exhibited a two-fold increase compared with the relative values in water, as shown in Figure 4b,c. Conversely, PC2.5 presented slightly reduced stiffness and toughness upon transitioning from water to PBS. Figure S3 shows the volume changes for NP, CMC gel, PAAm gel, DSPC, and PC2.5 observed by measuring the size of the samples. The results show that NP and CMC gels shrank in PBS after soaking for 168 h, with percentage volume changes of 50% and 90%, respectively. It is possible that the presence of ions in PBS could cause an alteration in the shielding of anionic groups (COO-) in CMC, increasing ion strength, decreasing osmotic pressure, and reducing the repulsion between polymer chains. As a result, the gel chains might move closer together, leading to shrinkage. PAAm gel, DSPC, and PC2.5 expanded slightly, with the percentage of volume change being less than 108%. The porous structure and high-density polyacrylamide might be expected to retain the distance between polymer chains, albeit changing the ion strength.

**Figure 5:**
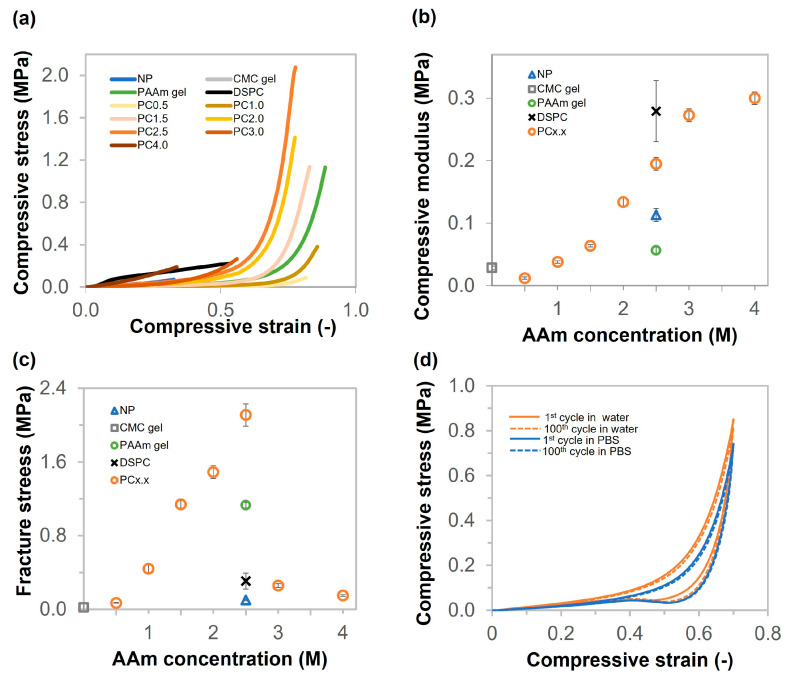
Compressive properties of NP, CMC gel, PAAm gel, DSPC, and PC gels (various AAm concentrations from 0.5 to 4 M). (**a**) Compressive curves of CMC gel, PAAm gel, NP, DSPC, and PC gels (various AAm concentrations from 0.5 to 4 M). (**b**,**c**) Compressive modulus and fracture stress values of CMC gel, PAAm gel, NP, DSPC, and PC gels (various AAm concentrations from 0.5 to 4 M). (**d**) Cyclic compressive curves of PC2.5 from 1 to 100 cycles in water and PBS.

The compressive stress–compressive strain curves of NP, CMC gel, PAAm gel, DSPC, and PC gels at various AAm concentrations and CMC 6 wt% are shown in Figure 5a. As shown in Figure 5b, the compressive moduli of CMC gel, PAAm gel, NP, and DSPC were 29, 57, 113, and 279 kPa, respectively. The compressive moduli of the PC gels increased from 12 to 300 kPa as the AAm concentration increased from 0.5 to 4.0 M. As described in Figure 5c, the compressive fracture stress values of CMC gel, NP, DSPC, and PAAm gel were 0.02, 0.10, 0.31, and 1.13 MPa, respectively. The compressive fracture stress of the PC gels increased from 0.07 to 2.11 MPa, prepared from AAm concentrations of 0.5 to 2.5. However, the fracture stress value dramatically decreased to 0.26 MPa when the AAm concentration exceeded 3.0 M. The compressive modulus and fracture stress of PC2.5 were approximately 6 and 105 times higher than those of NP, respectively. This indicates that the macropores in the porous gel are responsible for the mechanical increase. In addition, the Young’s modulus of DSPC was approximately 10 times higher than that of CMC gel due to high crosslinking density and high polymer content. The performance in terms of reusability was also evaluated using a cyclic compressive test. The cyclic compression results of PC2.5 in water and PBS are shown in Figure 5d. Successive compression tests performed on the porous gel between 0% and 70% strain indicate reversibility under both water and PBS conditions. The compressive moduli of PC2.5 in water and PBS were slightly reduced by approximately 5% and 10%, respectively, after 100 compression cycles. Thus, the porous gel can be compressed more than 100 times and immediately return to its original shape after unloading. Videos S2 and S3 illustrate the rapid elastic recovery of PC2.5 after undergoing folding and twisting deformations, respectively, highlighting its excellent shape-memory properties. However, DSPC was fractured after being folded and did not show the elastic property (Video S4).

In addition to evaluating the poroelastic behaviors of NP and PC2.5, compression tests with different compression rates (velocity) were performed in PBS. Figure S4a,b show the compression stress–strain curves of NP and PC2.5, respectively. As shown in Figure S4a, the compressive behavior of NP did not change significantly with the various compressive rates. On the other hand, as shown in Figure S4b, the compressive modulus of PC2.5 increased from 105 to 145 kPa when the compression rate was increased from 10 to 1000%/min. This phenomenon is a defining characteristic of poroelasticity, wherein the compressive stress depends on the rate of compression. The underlying mechanism is attributed to frictional interactions between the porous matrix walls and the interstitial fluid, which vary with the applied compression rate [40].

### 2.4. Physical Properties and Thermal Stability of NP, CMC Gel, PAAm Gel, DSPC, and PC Gels

**Figure 6:**
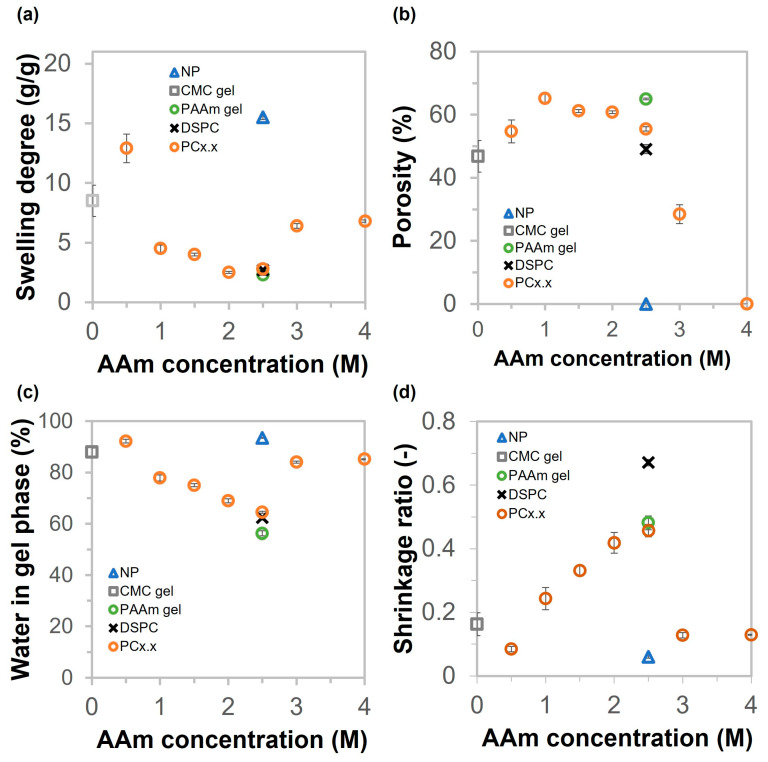
(**a**–**d**) Swelling behavior, porosity, water in the gel phase, and the shrinkage ratios of NP, CMC gel, PAAm gel, DSPC, and PC gels (various AAm concentrations from 0.5 to 4 M).

**Figure 7:**
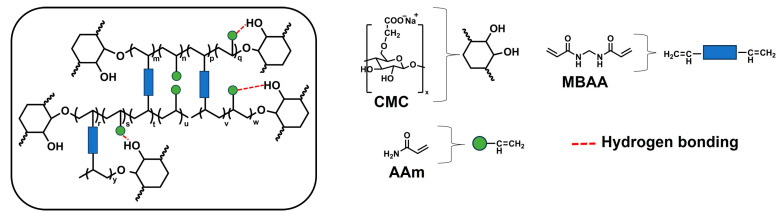
Schematic image of the chemical and physical bonding between polymers in PC2.5.

The swelling degree, porosity, water in the gel phase, and shrinkage of the hydrogels are shown in Figure 6. As illustrated in Figure 6a, the swelling degree values of NP, CMC gel, PAAm gel, and DSPC are 15.5, 8.5, 2.3, and 2.7 g/g, respectively. The swelling degrees of porous gels decreased steadily from 12.9 to 2.8 as the concentration of AAm increased from 0.5 to 2.5 M. After increasing the AAm concentration from 3.0 to 4.0 M, the swelling degrees recovered to 6.4 and 6.8 g/g, respectively. The porosity of hydrogels was determined by the amount of water in the pores divided by the swollen weight of hydrogels, as shown in Equation (2). The calculated results are shown in Figure 6b. The porosity of the porous gels, including CMC gel, PAAm gel, DSPC, and PAAm/CMC porous gels (PC gels), was relatively consistent, ranging from 49% to 65%. Notably, PC3.0 exhibited a relatively low porosity of approximately 29%. The NP and PC4.0 hydrogels have a non-macroporous structure. As shown in Figure 6c, the results of water content in the gel phase were similar to those of the swelling degrees. The water contents in the gel phase were 94%, 88%, 56%, and 62% for NP, CMC gel, PAAm gel, and DSPC, respectively. Analysis of PC gels showed that the water content in the gel phase decreased from 92% to 64% when prepared with AAm concentrations ranging from 0.5 to 2.5 M. The water contents in the gel phase of copolymer porous gels prepared with AAm concentrations of 3.0 and 4.0 M were 84% and 85%, respectively. The shrinkage ratios of NP, CMC gel, PAAm gel, DSPC, and PC gels were calculated as the dry volume divided by the swollen volume of the gel. The size of all swollen samples decreased after they were dried overnight in the oven, as shown in Figure 6d. The results showed that the shrinkage ratios of NP and DSPC were the lowest and highest, respectively. The shrinkage ratio of the PC gels continuously increased as AAm concentration increased from 0.5 M to 2.5 M; however, the shrinkage values of PC3.0 and PC4.0 were significantly reduced. The trends of the tensile modulus (Figure 3b) and shrinkage ratio (Figure 6d) were similar; it is possible that the highly dense polymer matrix at the pore wall inhibited the shrinkage of the porous gel. However, DSPC exhibited greater resistance to shrinkage compared with the other samples, likely due to its higher tensile and compressive moduli. These results indicate that the polymer matrix in DSPC was more rigid and formed a well-stabilized network, which effectively resisted structural collapse and thereby minimized shrinkage. Additionally, the hydrogen bond (H-bond) information of the gels was evaluated by comparing the changes in the volumes of gels in urea solution with those in water. Figure S5 shows the changes in the volumes of NP, CMC gel, PAAm gel, DSPC, and PC2.5. The results illustrate that the volume of the gels increased slightly after soaking overnight. It is possible that crosslinking density decreased because urea can disrupt intermolecular hydrogen bonding by forming competitive hydrogen bonds with polar functional groups, thus altering the structural organization of the polymer network. Based on the experimental results mentioned earlier, the structure of PC2.5 should exhibit both chemical and physical bonding, as shown in Figure 7.

**Figure 8:**
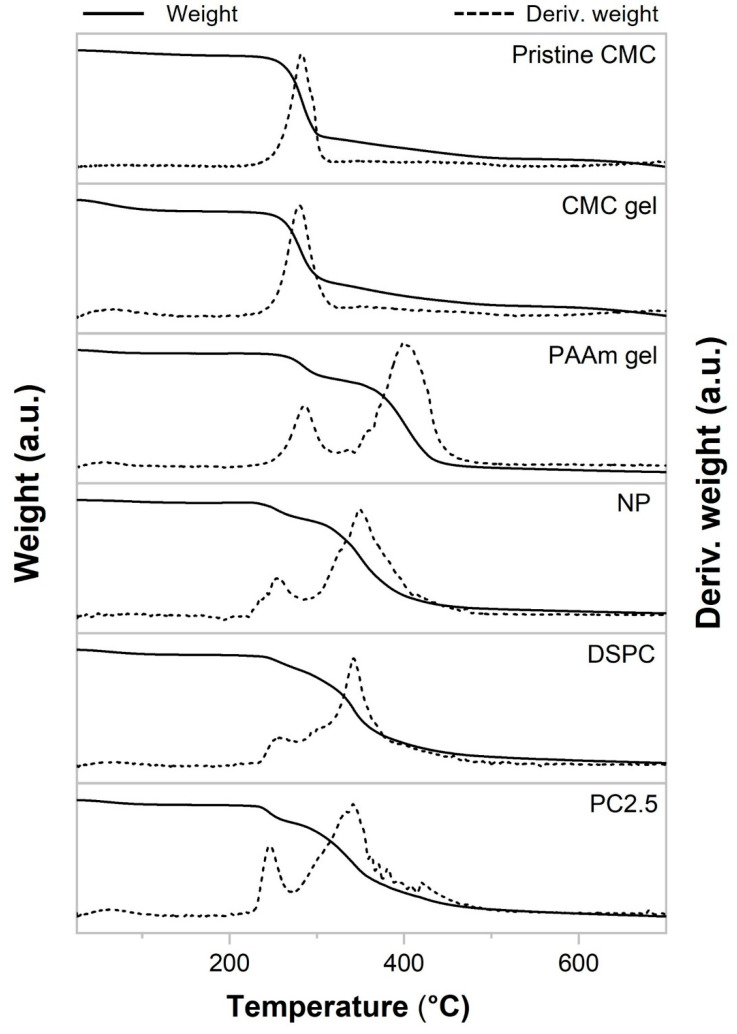
TGA and DTG thermal curves of pristine CMC, CMC gel, PAAm gel, NP, DSPC, and PC2.5.

Figure 8 presents the thermogravimetric (TGA) and derivative thermogravimetric (DTG) thermograms of pristine CMC, CMC gel, PAAm gel, NP, DSPC, and PC2.5. Water loss at the beginning of heating induced a decrease in weight. Water evaporation was completed at approximately 200 °C, and the water content values were below 6% for all samples. As the temperature continued to increase, decomposition began in all samples. The temperature range, temperature onset (Tonset), maximum decomposition temperature (Tmax) of DTG, and percentage of weight loss for each decomposition region of the samples are detailed in Table S2. The TG and DTG thermograms of all samples indicate one decomposition region for pristine CMC and CMC gel, while PAAm gel, NP, DSPC, and PC2.5 exhibit two decomposition regions. Pristine CMC began to decompose at 225 °C and continued until approximately 330 °C, with a maximum temperature (Tmax) observed at 266 °C, corresponding to a weight loss of approximately 48.0%. This was attributed to the decarboxylation of sodium CMC and the pyrolysis of the cellulose backbone [41]. After polymerization, the onset temperature and maximum temperature of the CMC gel were slightly lower than those of the original CMC powder. For PAAm gel, the first stage of decomposition, Tonset, and Tmax occurred at 225–325 °C, 266 °C, and 286 °C, respectively. This was due to the loss of pendant amide groups. The majority of the weight loss (56.3%) occurred in the second stage of decomposition (340–500 °C), with a Tmax of approximately 401 °C, which was attributed to the decomposition of the main chain [42]. The gels produced by combining CMC and acrylamide are NP, DSPC, and PC2.5, and their primary decomposition occurred in the second stage. The Tonset and Tmax for NP, DSPC, and PC2.5 were 322 and 349 °C, 323 and 343 °C, and 309 and 343 °C, respectively. Their thermal stabilities were reduced compared with the PAAm gel and the two-step decomposition process nearly overlapped, especially for DSPC.

### 2.5. Drug Release Profiles of CMC Gel, PAAm Gel, NP, DSPC, and PC Gel

**Figure 9:**
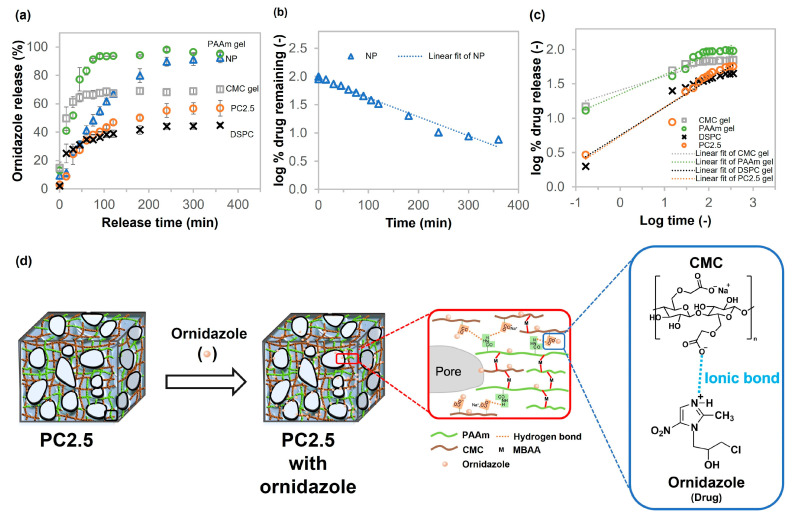
(**a**) Ornidazole release behaviors of NP, CMC gel, PAAm gel, DSPC, and PC2.5, (**b**) first-order and (**c**) Korsmeyer–Peppas models for analyzing drug release behavior, and (**d**) internal structure of PC gel and interaction between ornidazole and CMC.

The developed porous gels are intended for biomaterial engineering applications, which often involve material implantation or wound healing scenarios carrying a high risk of infection. Consequently, selecting an antibiotic agent as a model drug directly aligns with the intended practical applications of these materials. When selecting an antimicrobial agent suitable for incorporation into a drug delivery system based on a hydrogel composed of polyacrylamide (PAAm) and carboxymethyl cellulose (CMC), it is crucial to consider the physicochemical properties of the drug to ensure compatibility and sustained release performance. Ideal candidates should possess aqueous solubility and the potential for molecular interactions with the polymeric network. Ornidazole, a nitroimidazole-class antimicrobial agent, possesses suitable properties for use as a model drug due to its water solubility and polar functional groups, including hydroxyl, nitro, and imidazole groups. The polar functional groups enable potential non-covalent interactions with the amide groups of PAAm and the carboxylate groups of CMC, enhancing drug loading and sustained release. Additionally, literature reviews report ornidazole release from other polyacrylamide-based hydrogels with polysaccharides, such as dextran and hydroxypropyl methyl cellulose [43,44]. This supports its suitability as a model compound in this study. The drug release profiles of NP, CMC gel, PAAm gel, DSPC, and PC2.5 are shown in Figure 9. All of the samples could adsorb and release ornidazole, which acts as an antibiotic. The results in Figure 9a show that the drug was released rapidly (within 90 min) in the early stage. The release rate then slowed, stabilizing at 360 min. NP and PAAm gels released almost 100% of the ornidazole. The CMC gel, DSPC, and PC2.5 had released all of the ornidazole at approximately 70 min, 45 min, and 57 min, respectively. The diffusion time constant, determined by the sample release rate, was calculated for the early stage of ornidazole release from 0 to 90 min, as illustrated in Figure S6a. PAAm gel released the antibiotic agent faster than other samples, with a diffusion time constant of 32 min. The CMC gel showed a slower release rate than the PAAm gel (approximately 2.7 times slower). In addition, the Ikeuba team analyzed the molecular electrostatic potential (MESP) surface of ornidazole to predict its reactive sites for electrophilic attack. The NH was located in electron-poor regions (positive charge), indicating that this part of the molecule was prone to electrophilic attack [45]. We expected that the COO- of CMC molecules could interact with the NH of ornidazole through ionic bonds shown in Figure 9d. Therefore, the diffusion time constant of PAAm gel was the lowest compared with NP, PC2.5, and DSPC (composed of CMC) of 123, 182, and 250 min, respectively. Furthermore, the drug release data were fitted using four kinetic models: First-order, Korsmeyer–Peppas, Zero-order, and Higuchi to obtain the release mechanisms of the drugs, as shown in Figure 9b,c and Figure S6b,c, respectively.

For the kinetic model, the equations were as follows [46,47]:

Zero-order release kinetics: W = k_0_t, where W is the percentage of drug released at time t, k_0_ is the zero-order release constant, and t is the release time.

First-order kinetics: log (100 − W) = k_1_t/2.303, where k_1_ is the first-order release constant.

Higuchi model: W = k_H_t^1/2^, where k_H_ is the Higuchi constant,

Korsmeyer–Peppas model: Mt/Mα = k_KP_t^n^, where Mt/Mα is the fraction of drug release at time t, k_KP_ is the Korsmeyer–Peppas constant, and n is the diffusion exponent, which indicates the transport mechanism.

The best-fitting model of drug release was determined by evaluating and comparing the correlation (R^2^) values of the aforementioned models, the results of which are shown in Table 2. A comparison of the R^2^ values of different models indicates that the R^2^ value of the Korsmeyer–Peppas model for porous gels (CMC gel, PAAm gel, DSPC, and PC2.5) is higher than that of other kinetic models. The release mechanism was determined by considering the n value: *n* ≤ 0.5 means that the mechanism corresponds to a Fickian diffusion; at 0.5 ≤ *n* ≤ 1.0, the mechanism approaches anomalous (non-Fickian) diffusion; and *n* = 1.0 shows that the release mechanism approaches Case II transport. In this study, the n values for CMC gel, PAAm gel, DSPC, and PC2.5 were 0.20, 0.28, 0.40, and 0.42, respectively, which were less than 0.5. This suggests that the release of ornidazole from porous gels was mainly mediated by Fickian diffusion. This mechanism allows the drug to diffuse through the swollen polymer matrix and through water-filled pores [48]. Additionally, the results indicated that the drug release from the NP fitted the first-order model. This means that the drug release behavior depends on concentration [49]. Furthermore, both NP and PC2.5 exhibited increasing HFLS cell ratios, with a statistically insignificant difference (*p* ≥ 0.05) according to the one-way analysis of variance (ANOVA) test, as illustrated in Figure S7. After cell culture, the remaining cells in the hydrogels continued to grow regularly in the culture medium; therefore, neither the non-cryo gel nor the porous gels were toxic to cells.

## 3. Conclusions

A polyacrylamide/carboxymethyl cellulose porous gel (PC gel) was successfully prepared via one-pot cryogelation with free radical polymerization. This approach offers a more accessible and efficient pathway to achieving covalent crosslinking in CMC-based hydrogels without relying on harsh conditions, organic solvents, or chemically modified cellulose. The cryogelation technique can produce a highly dense polymer at the pore wall, in macropores, and within an interconnected porous structure. Thus, the PC gel (copolymer network) and DSPC gel (interpenetrated network) exhibited higher mechanical properties than the single-porous gels (CMC gel and PAAm gel) and the non-cryo gel (NP). The experimental results demonstrated that the two substances and the cryo-process significantly improved the mechanical properties of the gels. Furthermore, the PC gel exhibited the highest toughness when prepared using 2.5 M AAm, 6 wt% CMC, 1 mol% crosslinker, and 0.1 mol% initiator. The morphology of the PC gel was that of an elastic sponge (i.e., it fills with water and solvent and returns to its original shape after being squeezed). The mechanical properties and shape of PC2.5 were not significantly different when tested in different solutions (pure water and PBS). Regarding the cyclic compression test, the modulus of PC2.5 decreased slightly after 100 compressions under wet conditions. Finally, PC2.5 exhibited slower drug release than NP, despite its components being similar to those of NP. The release behavior of PC2.5 was best described using the Korsmeyer–Peppas model with a predominant mechanism of Fickian diffusion. Neither PC2.5 nor NP was toxic to cells; therefore, the PC gel shows good potential for bioengineering applications.

## 4. Materials and Methods

### 4.1. Materials

Acrylamide (AAm) was purchased from Junsei Chemical Co., Ltd. (Kyoto, Japan). Sodium carboxymethyl cellulose (CMC), molecular weight = 100,000–110,000 g/mol, N, N′-methylenebisacrylamide (MBAA), 2-oxoglutaric acid (α-keto), and 0.25 w/v% trypsin solution were bought from Fujifilm Wako Pure Chemical Corporation (Osaka, Japan). Ornidazole (Orn; >98.0%), used as an antibiotic, was obtained from Tokyo Chemical Industry (Tokyo, Japan). Human fibroblast-like synoviocytes (HFLSs) were obtained from normal, healthy human synovial tissues and purchased from Cell Applications (San Diego, CA, USA). Trypan blue staining was purchased from Thermo Fisher Scientific (Waltham, MA, USA). Phosphate-buffered saline (PBS) and culture medium were provided by Nacalai Tesque, INC. (Kyoto, Japan), and Sigma-Aldrich (St. Louis, MO, USA), respectively. All chemicals were analytical grade and used as received.

### 4.2. Preparation of PAAm/CMC Porous Gels

**Figure 10:**
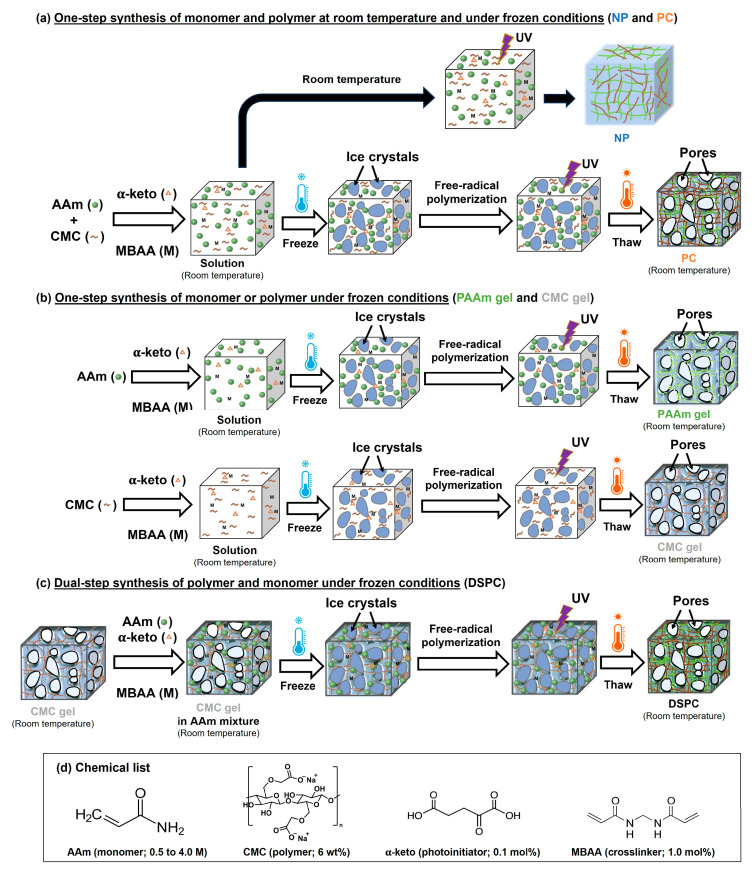
Scheme of (**a**) one-pot synthesis of monomer and polymer porous gel at room temperature under freezing conditions, (**b**) one-pot synthesis of monomer or polymer porous gel under freezing conditions, (**c**) two-step synthesis of polymer and monomer porous gel under freezing conditions, and (**d**) chemical list.

PAAm/CMC porous gels (PC gels) were prepared using a simple one-pot preparation procedure initiated by ultraviolet (UV) polymerization under freezing conditions. AAm, CMC (6 wt%), MBAA (1 mol% to AAm), and α-keto (0.1 mol% to AAm) were dissolved in pure water. The precursor solution was poured into a glass mold consisting of two glass plates with a 3 mm silicone rubber spacer. After this, the precursor in the mold was frozen at −16 °C for 2 h in an ethanol cooling bath (NCB-3300, Tokyo Rikakikai Co., Ltd., Tokyo, Japan) to promote high-yield crosslinking polymerization. Polymerization was then performed under 365 nm UV irradiation. After polymerization for 20 h, the freezing solution in the mold was extracted from the cooling bath and thawed at room temperature (25 °C). The hydrogels with different concentrations of AAm (0.5, 1.0, 1.5, 2.0, 2.5, 3.0, and 4.0 M), with CMC at 6 wt%, were named PC0.5, PC1.0, PC1.5, PC2.0, PC2.5, PC3.0, and PC4.0, respectively. The non-cryo gel was prepared using the same method as PC2.5, but the polymerization conditions were room temperature and an argon gas atmosphere. In addition, the CMC gel and the PAAm gel were prepared as described above, except that AAm and CMC were added, respectively. For the double synthesis process (DSPC), the porous gel was prepared via a two-step procedure using UV-initiated polymerization under freezing conditions. CMC gel is the first network for DSPC. The CMC gel was soaked in excess aqueous solution containing the AAm mixture, which had the same composition as the PAAm gel. The CMC gel with the mixture precursor solution was frozen at −16 °C for 2 h and then UV-irradiated. The subsequent steps were performed in the same manner as described earlier. All hydrogel samples were washed with pure water and soaked in pure water for at least 7 days before characterization. The scheme for all hydrogels, along with the details of the components and conditions for the samples, is shown in Figure 10.

### 4.3. Characterization

Fourier transform infrared (FTIR) spectra were measured using an attenuated total reflectance (ATR) FTIR spectrometer (FT/IR-6600, JASCO Corporation, Hachioji, Japan) at a resolution of 4 cm^−1^, 64 scans, and the spectral range of 4000–400 cm^−1^ for each sample. The morphologies of the hydrogels were observed using the 3D laser scanning microscope (VK9710, KEYENCE CORPORATION, Osaka, Japan). Thermogravimetric analysis (TGA) was performed on a Thermo plus EVO II TG8120 HUM-1F (Rigaku Corporation, Akishima, Japan) under nitrogen gas with a heating rate of 10 °C/min.

### 4.4. Mechanical Tests

A tensile test was performed on square swollen hydrogels (8 mm × 50 mm) using a universal machine (Instron 5965, Instron Co., Norwood, MA, USA) at room temperature (25 °C). The mechanical properties of the hydrogels were measured at a strain rate of 10 mm/min for the tensile test. Tensile modulus and toughness were obtained from the tensile stress–strain curve. The tensile modulus was calculated from the slope at strains from 0.01 to 0.1, and the toughness was calculated from the area of the tensile stress–strain curve.

Compression and cyclic compression tests were performed for the hydrogels using Tensilon RCT 1310A and RTG-1310 (A&D Manufacturing Company, Limited, Tokyo, Japan), respectively, at room temperature (25 °C). The diameter of the swollen cylindrical hydrogel was 10 mm. For the compression test, the strain rate was kept constant at 10%/min. All moduli of the samples were calculated from the slope at the beginning of the compressive stress–strain curve. For the cyclic compression test, the PC2.5 specimen was tested in water and PBS at room temperature. Prior to testing in PBS, the PC2.5 was soaked in PBS, and the buffer was changed daily for three days. The compression rate was 100%/min. The number of compressive cyclic loading–unloading measurements was one hundred. NP and PC2.5 were also compressed in the PBS at compression rates of 10, 100, and 1000%/min to observe their elastic properties. Before testing, they were soaked in the buffer for over 3 days, with fresh PBS added daily.

### 4.5. Swelling Behavior, Porosity, and Water in the Gel Phase

#### 4.5.1. Swelling Degree

The swelling degree of the porous gels was determined by the change in the weight of the squeezed hydrogel (*W*_sq_; water squeezed out) and the dried hydrogel (*W*_d_). The water in the pores was removed by compressing the swollen porous gel. The compression force of the swollen porous gels was used for about 95% of the fracture stress. The swelling degree was calculated as follows:

(1) $$Swelling\;degree=\frac{Wsq}{Wd}$$

#### 4.5.2. Porosity

The porosity (*P%*) of the porous gels was measured using the swollen sample weight (*W_s_*) and the sample weight after compressing to squeeze out the water in the pores (*W_sq_*). The sample was squeezed three times between tissue papers and then immediately weighed [36].

(2) $$Porosity\;(P\%)=(1-\frac{Wsq}{Ws})\times 100$$

#### 4.5.3. Water Content in the Gel Phase

After the porosity measurement test, the porous gels were dried in an oven (80 °C) until their weight remained constant (*W_d_*). Then, the water content in the gel phase was calculated as follows [36].

(3) $$Water\;content\;in\;the\;gel\;phase\;(\%)=100\times (1-\frac{Wd}{Wsq})$$

#### 4.5.4. Drug Release Test

The experimental conditions (including temperature and type of solvent) were modified based on the setup described in the reference [43]. The drug release profile of each gel was evaluated by measuring the amount of released drug in the PBS solution compared with the total drug in the gel at different time points. The dried sample was kept in the 8 mg/mL ornidazole aqueous solution for 24 h; then, the sample was dried at 35 °C and weighed. The loaded drug in the gel was calculated as the difference between the initial dried weight of the gel (before drug loading) and the dried weight after drug loading. The drug-loaded gel was soaked in 240 mL PBS solution (pH = 7.2–7.4) at 32 °C and gently shaken continuously in a shaking incubator (PIC-100S, AS ONE, Osaka, Japan) at approximately 60 rpm to reach equilibrium early. The drug release profiles of all samples were measured in triplicate to ensure reproducibility. Then, 0.8 mL aliquots were taken at different time intervals and characterized using a UV-Vis spectrophotometer (UV-1800, Shimadzu Corporation, Kyoto, Japan). After measurement, the aliquot was returned to the container. The quantity of cumulative drug release was calculated as follows:

(4) $$Cumulative\;drug\;release(\%)=\frac{m_{t}}{m_{0}}$$

where *m*_t_ is the cumulative mass of ornidazole released at an interval time, and *m*_0_ is the total amount of ornidazole in the hydrogel. The release rate of ornidazole from the gels was determined using the diffusion time constant (*τ*). The diffusion time constant was calculated as follows [50]:

(5) $$I=I_{max}(1-e^{-t/\tau })$$

where *I* is the absorbance value of ornidazole released at an interval time, *I*_max_ is the absorbance value reached at a constant maximum value, and *t* is the interval time.

### 4.6. Cytotoxicity Test

The cytotoxicity of the gels was evaluated against human fibroblast cells (HFLSs). The samples were washed twice with PBS and then sterilized in PBS using an autoclave (Labo autoclave, Sanyo, Osaka, Japan). The sterilized gels were immersed in the culture medium and placed on a well-culture plate. Then, HFLSs were seeded on the gels and incubated under 5% CO_2_ at 37 °C for 5 days. The culture medium was refreshed once on the third day of incubation. After incubation, gels with cells were washed twice with PBS to remove the dead cells. Then, trypsin was added to each well to extract live cells from each sample, and the solution was collected in a tube. The samples were washed with PBS, and the solution was kept in the previous tube. The cells in the solution were separated by centrifugation (2000 rpm, 5 min) and washed again with PBS. Finally, the live HFLS cells in the PBS were stained with trypan blue stain and monitored using a counter machine (Countess 3, Invitrogen, Waltham, MA, USA) to count the number of cells. The increasing live-cell number for all samples showed a statistically insignificant difference (*p* ≥ 0.05), according to the one-way analysis of variance (ANOVA) test. This was followed by the Bonferroni multiple comparison procedure (OriginPro 2023 software, OriginLab, Northampton, MA, USA).

## Figures and Tables

**Table 1:** Comparative fabrication of CMC-based hydrogels via free radical polymerization induced by external energy sources.

Energy Type and Source	CMC Concentration (W/V) %	Requirement	Solvent	Yield * (%)	Ref
Ionizing radiation	0.5	- Low pH, - Oxygen removal	- Water	70	[26]
Gamma ray	30	- Oxygen removal - Vacuum system	- Water	82	[27]
Gamma ray	15	- MBAA (crosslinker)	- Water	~60	[24]
UV lamp	5	- Modified dialdehyde CMC - Preparation of cinnamic acid hydrazide (Crosslinker) (prepared from cinnamoyl chloride)	- Acetone - Dimethyl formamide, - Acetonitrile - Ethanol	75	[28]
UV lamp (10 mW/cm^2^)	1	- Functionalization of CMC with norbornene - 2,2′-(ethylenedioxy) diethanethiol (crosslinker) - Irgacure 2959 (photoinitiator)	- PBS	No data	[29]
Light-emitting diode light (30 mW/cm^2^) A medium-pressure mercury lamp with UV lamp (12 mW/cm^2^) (post-curing)	2	- Functionalization of CMC with methacrylic anhydride - Lithium phenyl-2,4,6-trimethyl benzoylphosphinate (initiator)	- Water	87	[30]
UV lamp	1	- Modified CMC using lysine and allyl glycidyl ether (CLA) - MBAA (crosslinker) - Riboflavin (initiator) - Potassium persulfate (co-initiator)	- pH 5.7 (dissolved CLA) - PBS	~95	[31]
UV lamp (1.0 mW/cm^2^)	6	- Ethanol bath (freezing) - MBAA (crosslinker) - α-keto (initiator)	- Water	~99	This work

* The percent yield was calculated by dividing the weight of the dry sample after washing by the weight of the dry sample before washing.

**Table 2:** Various kinetic data for NP, CMC gel, PAAm gel, DSPC, and PC2.5.

Sample Name		Kinetic Model			Remark
	Zero Order	First Order	Higuchi	Korsmeyer–Peppas	
	R^2^	R^2^	R^2^	R^2^	n
NP	0.8399	0.9763	0.9623	0.8018	0.34
CMC gel	0.2949	0.3621	0.5817	0.9003	0.20
PAAm gel	0.4379	0.6478	0.7316	0.9379	0.28
DSPC	0.5302	0.6090	0.8094	0.9318	0.40
PC2.5	0.7168	0.8093	0.9266	0.9369	0.42

## Data Availability

The original contributions presented in this study are included in the article/Supplementary Materials. Further inquiries can be directed to the corresponding author.

## Supplementary Materials

The following supporting information can be downloaded at: https://www.mdpi.com/article/10.3390/gels11060453/s1, Table S1: The details of CMC-based sample conditions, except CMC gel; Figure S1: Images before and after swollen gel in the water of C4 sample prepared using high CMC concentration, crosslinker, and initiator; Figure S2: Stress–strain curves of as-prepared NP and as-prepared PC2.5; Figure S3: Volume changes for NP, CMC gel, PAAm gel, DSPC, and PC2.5 in PBS compared with those in water; Figure S4: (a,b) The compression stress–strain curves of NP and PC2.5 with various compression rates (velocity) in PBS at room temperature, respectively; Figure S5: Volume changes for NP, CMC gel, PAAm gel, DSPC, and PC2.5 in urea solution compared with those in water; Table S2: The temperature range, temperature onset (Tonset), maximum decomposition temperature (Tmax) of DTG, and percentage weight loss for each decomposition region of the samples.; Figure S6: (a) Drug concentration released of NP, CMC gel, PAAm gel, DSPC, and PC2.5 as a function of time (release time 0–90 min), (b) zero-order, and (c) Higuchi models for analyzing drug release behavior.; Figure S7. The ratio of the cell increases after incubation in Control (No hydrogel), NP, and PC2.5. Results identify the mean value ± standard error; Video S1: PC2.5 behavior during the squeezing; Video S2: PC2.5 behavior during the folding test; Video S3: PC2.5 behavior during the twisting test; Video S4: DSPC behavior during the folding test.

## Abbreviations

The following abbreviations are used in this manuscript:

AAm	Acrylamide
PAAm	Polyacrylamide
CMC	Carboxymethyl cellulose
MBAA	N, N’-methylenebisacrylamide
α-keto	2-oxoglutaric acid
NP	PAAm/CMC non-cryo gel
PC	PAAm/CMC porous gel
DSPC	Dual-step synthesis porous gel
CMC gel	CMC porous gel
PAAm gel	PAAm porous gel
PBS	Phosphate-buffered saline
FTIR	Fourier transform infrared
