# Supplementary material for: Porous and Tough Polyacrylamide/Carboxymethyl Cellulose Gels Chemically Crosslinked via Cryo-UV Polymerization for Sustained Drug Release
Source: Gels. 2025 Jun 13;11(6):453. doi: 10.3390/gels11060453 (PMC12191759; doi:10.3390/gels11060453)
Supplement: Supplementary file 1 [file gels-11-00453-s001.zip › gels-3662275-supplementary.pdf]

Supporting Information

of

Porous and Tough Polyacrylamide/Carboxymethyl Cellulose Gels Chemically Crosslinked via Cryo-UV Polymerization for Sustained Drug Release

Duangkamon Viboonratanasri[1], Daniel Rudolf King[2†], Tsuyoshi Okumura[2], Mohamad Alaa Terkawi[3], Yoshinori Katsuyama[2], Milena Lama[4], Tomoki Yasui[2], Takayuki Kurokawa[2,*]

[1] Graduate School of Life Science, Hokkaido University, Sapporo 001-0021, Japan; viboonratanasri.duangkamon.j9@elms.hokudai.ac.jp
[2] Faculty of Advanced Life Science, Hokkaido University, Sapporo 001-0021, Japan; dking@sci.hokudai.ac.jp (D.R.K.); tsuyoshi.okiami@gmail.com (T.O.); y.katsuyama@sci.hokudai.ac.jp (Y.K.); tyasui@sci.hokudai.ac.jp (T.Y.)
[3] Faculty of Medicine and Graduate School of Medicine, Hokkaido University, Sapporo 060-8638, Japan; materkawi@med.hokudai.ac.jp
[4] Institute for Chemical Reaction Design and Discovery (WPI-ICReDD), Hokkaido University, Sapporo 001-0021, Japan; lama@sci.hokudai.ac.jp
* Correspondence: kurokawa@sci.hokudai.ac.jp
† Deceased 1 May 2022

In this work, the effect of cryoconcentration on the polymerization reaction was investigated, as shown in Table S1. Interestingly, we found that chemical concentration and UV irradiation were important factors for polymerization. C1 sample was prepared with a low mixture concentration at room temperature (25 °C) with UV irradiation, and after irradiation, C1 sample was still a solution (not a gel). The mixture concentration of C2 sample was similar to that of C1 sample, but the conditions of C2 sample were frozen without UV irradiation. After the preparation, the viscosity of C2 samples increased, but it was still dissolved in water. Then, C3 sample was prepared with only frozen low CMC concentration (without both crosslinker and initiator). It became a viscous solution and dissolved in water. The C3 sample was then prepared using only the frozen low CMC concentration (without both crosslinker and initiator). It became a viscous solution and dissolved in water. In addition, we successfully prepared the non-cryo CMC hydrogel (Fig. S1) via a high-mixture solution at room temperature with UV irradiation, which is indicative of C4. The gel did not dissolve in water, and the swelling ratio was about 450 (calculated by the swollen weight of the gel divided by the dried weight of the gel). The indentation test described the mechanical property of the C4 sample, as the gel was very brittle. The indentation modulus of swollen C4 gel was 1.4 ± 0.1 kPa. In the case of C5 samples, the mixture concentration of C5 sample was the same as that of C4, but the mixture of C5 was frozen without UV irradiation. After freezing, C5 became solid and then dissolved in water. Hara's team reported that the CMC gels prepared with gamma rays depended on the CMC concentration, an important parameter in CMC gel preparation. That is, when the CMC concentration was low, the gel was still in the liquid state after irradiation, whereas when the CMC concentration was high, some chunks were found in the irradiated sample [1]. In addition, we attempted to increase the energy to induce radical generation by using a hyper UV lamp, the power of which was approximately 200 times higher than that of the standard UV lamp. The initial concentration of the sample was similar to C1, identified as the C6 sample. After UV irradiation, the sample formed a viscous solution, which was then dissolved in water. In addition, we performed two other conditions: only a low CMC concentration (C7 sample) and CMC with the initiator (C8 sample). Each sample was still in the liquid state in the frozen irradiated samples.

Table S1 The details of CMC-based sample conditions, except CMC gel.

Sample name	Temperature (℃)	UV irradiation	CMC concentration (wt%)	MBAA	**α**-keto	Texture (as prepared)	Dissolved in water
C1	25	Yes	6	Yes	Yes	Solution	Yes
C2	Frozen	No	6	Yes	Yes	Viscous solution	Yes
C3	Frozen	No	6	No	No	Viscous solution	Yes
C4	25	Yes	15	Yes	Yes	Solid	No (Hydrogel)
C5	Frozen	No	15	Yes	Yes	Slurry	Yes
C6	25	Hyper UV	6	Yes	Yes	Viscous solution	Yes
C7	Frozen	Yes	6	No	No	Viscous solution	Yes
C8	Frozen	Yes	6	No	Yes	Viscous solution	Yes

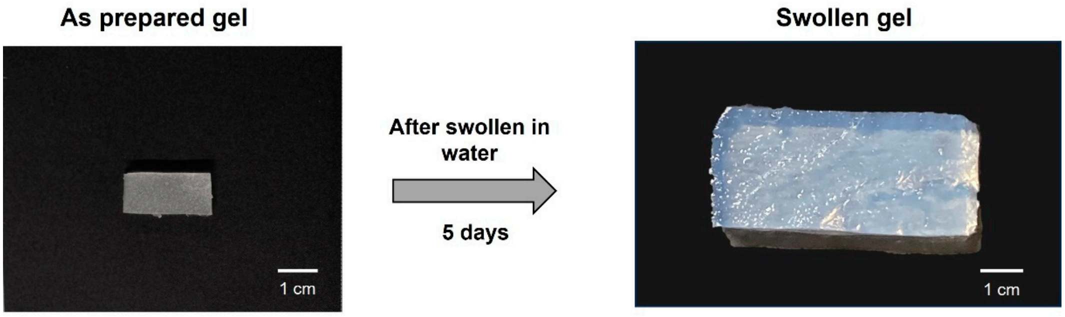

Figure S1. Images before and after swollen gel in the water of C4 sample prepared using high CMC concentration, crosslinker, and initiator.

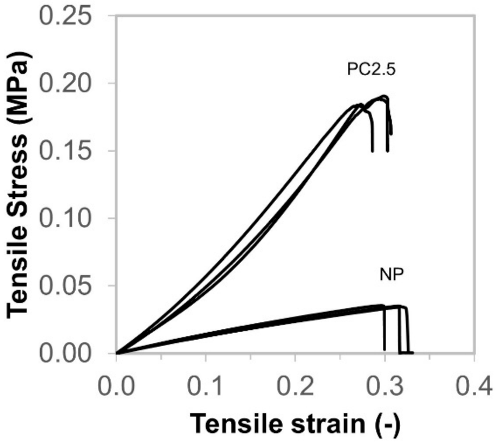

Figure S2. Stress-strain curves of as-prepared NP and as-prepared PC2.5.

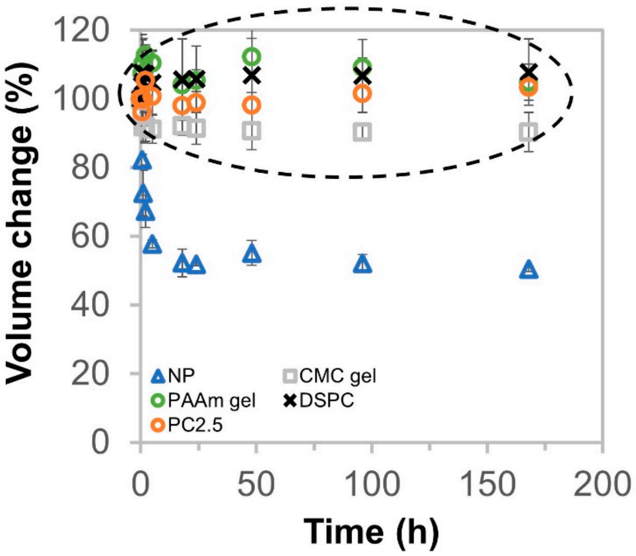

Figure S3. Volume changes of NP, CMC gel, PAAm gel, DSPC, and PC2.5 in PBS compared with those in water.

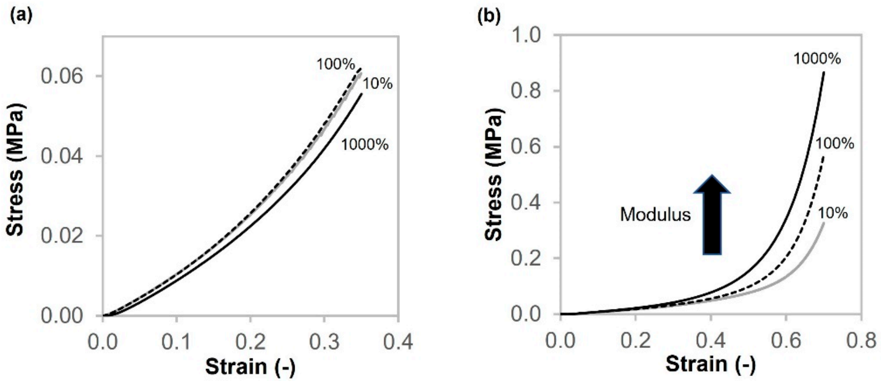

Figure S4. (a,b) The compression stress-strain curves of NP and PC2.5 with various compression rates (velocity) in PBS at room temperature, respectively.

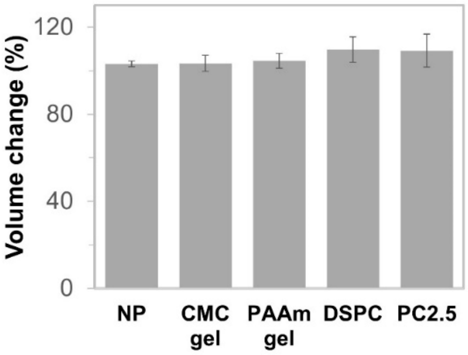

Figure S5. Volume changes of NP, CMC gel, PAAm gel, DSPC, and PC2.5 in urea solution compared with those in water.

Table S2. The temperature range, temperature onset (Tonset), maximum decomposition temperature (Tmax) of DTG, and percentage weight loss for each decomposition region of the samples.

Sample name	Decomposition region	Temperature range (°C)	Tonset (°C)	Tmax (°C)	Weight loss (%)
**Pristine CMC**	I	225-330	266	283	48.0
**CMC gel**	I	225-330	261	280	34.9
**PAAm gel**	I	225-325	266	286	17.3
	II	340-500	373	401	56.3
**NP**	I	215-290	237	255	12.2
	II	290-500	322	349	61.3
**DSPC**	I	200-280	241	255	10.8
	II	280-500	323	343	62.4
**PC2.5**	I	225-270	236	246	11.3
	II	270-500	309	343	60.11

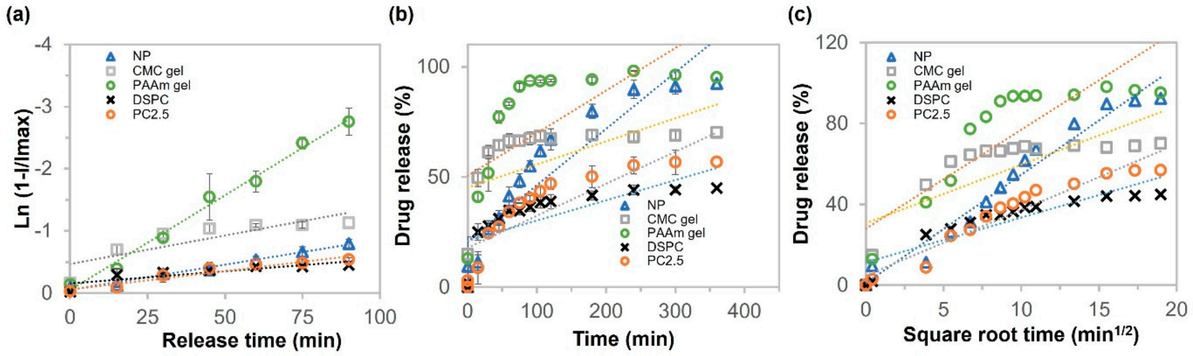

Figure S6. (a) Drug concentration released of NP, CMC gel, PAAm gel, DSPC, and PC2.5 as a function of time (release time 0-90 min), (b) zero-order, and (c) Higuchi models for analyzing drug release behavior.

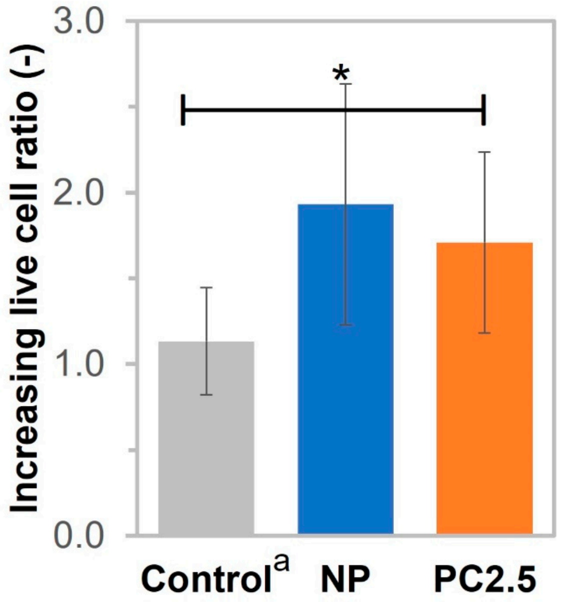

Figure S7. The ratio of the cell increases after incubation in Control (No hydrogel), NP, and PC2.5. Results identify the mean value ± standard error.

* represents a significant difference as determined by one-way analysis ($p \geq 0.05$), and [a] represents the control sample, tested without hydrogel. Statistical data was used in the one-way analysis of variance (ANOVA) test, followed by the Bonferroni multiple comparison procedure (OriginPro 2023 software, USA).

Video S1: PC2.5 behavior during the squeezing

Video S2: PC2.5 behavior during the folding test

Video S3: PC2.5 behavior during the twisting test

Video S4: DSPC behavior during the folding test.

References

1. Hara, K.; Iida, M.; Yano, K.; Nishida, T. Metal Ion Absorption of Carboxymethylcellulose Gel Formed by γ-Ray Irradiation. *Colloids Surf B Biointerfaces* **2004**, *38*, 227–230
